# Nurse-Led Education and Engagement for Diabetes Care in Sub-Saharan Africa: Protocol for a Mixed Methods Study

**DOI:** 10.2196/15408

**Published:** 2020-06-03

**Authors:** Arti Singh, Michelle Nichols

**Affiliations:** 1 University Hospital Kwame Nkrumah University of Science and Technology Kumasi Ghana; 2 College of Nursing Medical University of South Carolina Charleston, SC United States

**Keywords:** diabetes, mobile health, Ghana, sub-Saharan Africa, global health, nurses, task-shifting, mixed methods, focused ethnography

## Abstract

**Background:**

As the impact of diabetes grows steeply in sub-Saharan Africa, improvement of the control and treatment of diabetes is a goal that health care systems in sub-Saharan Africa must achieve in the near future. Sub-Saharan Africa faces a number of challenges in addressing the increasing effects of diabetes. One important factor is the shortage of adequately trained health care workers. Diabetes management in sub-Saharan Africa would benefit from innovative approaches that are founded upon solid theoretical constructs, built upon existing human resources and infrastructure, and culturally tailored to the priorities and needs of the local population. Existing resources, such as mobile phones and task-shifting strategies, may be used to assist individuals with glycemic self-management and to facilitate management of additional day-to-day clinical responsibilities.

**Objective:**

The objective of the Nurse-Led Education and Engagement Study for Diabetes Care (NEEDS) mixed-methods protocol is to develop a practical, collaborative, effective, and sustainable program for diabetes prevention and management specifically for patients with type 2 diabetes mellitus in sub-Saharan Africa. The protocol aims to improve access to care through task-shifting strategies and the use of mobile health technology.

**Methods:**

This study was designed using a convergent parallel mixed-methods approach that consisted of surveys, key informant interviews, focus group discussions, and focused ethnography. Novel approaches, such as task-shifting strategies and the use of mobile technology, were implemented for type 2 diabetes mellitus health care in sub-Saharan Africa—currently an under-researched area.

**Results:**

Data collection began in February 2018, after ethics approval, at the Kwame Nkrumah University of Science and Technology. As of May 2020, participant surveys have been completed (N=100), key informant interviews (n=7) have been completed, and focus groups (5 focus groups; patients, n=18; caregivers, n=6; community leaders, n=2; and faith leaders, n=3) as well as focused ethnographic field observations have been completed. All audio recordings have been transcribed and transcripts of sessions recorded in Twi have been translated to English. Data analysis is currently underway and anticipated completion is in the spring of 2020. Following data analysis, investigators plan to publish study findings.

**Conclusions:**

Insights from this study will inform the preliminary development of a feasible and effective nurse-led education and engagement mobile health intervention that has the potential to reduce diabetes-related morbidity, mortality, and burden in sub-Saharan Africa.

**International Registered Report Identifier (IRRID):**

DERR1-10.2196/15408

## Introduction

### Background

Diabetes is a global public health concern [1]. Africa, which is home to 49 diverse sub-Saharan African territories, had an estimated 15.5 million people living with diabetes in 2017 [2], and it is expected that number will increase to 40.7 million by 2045 [3]. Sub-Saharan Africa has the highest proportion of undiagnosed cases of diabetes (69.2%) and the highest complication rates compared to high-income countries [4]. Approximately 6% of mortality in Africa is attributed to diabetes and the highest all-cause mortality due to diabetes occurs among individuals aged 30 to 39 [4]. It has also been reported that over 90% of people in sub-Saharan Africa with diabetes have type 2 diabetes [4], and less than 40% of those living with the condition maintain optimal glycemic control [5].

The social, political, and economic constitution of sub-Saharan Africa is hugely diverse, as are its countries’ health systems. Diabetes has a major economic impact at both the personal and national level [4,6]. Political instability, poor health literacy, limited health budgets, limited facilities, inadequate clinical expertise and personnel, poor drug supply, out-of-pocket health expenditures, minimal health insurance systems, and individual behavior factors are the most common challenges faced by health care systems in sub-Saharan Africa [4,6]. The rising prevalence of diabetes and its associated comorbidities also impose an increasing financial burden on health care systems. Many treatments for diabetes are too expensive for people in sub-Saharan Africa to afford [5]. Despite the high cost of managing diabetes, health care professionals in sub-Saharan Africa must often be more focused on acute medical treatment rather than on prevention; existing health care systems often lack adequate policies and guidelines to address the gap in needs [7]. Furthermore, poor diabetes management and self-care in the region have been associated with “healer shopping”—alternating between traditional providers and faith healers [8].

Major concerns within sub-Saharan Africa include a shortage in the number of health care workers and inadequate training of health care workers, both which have led to a crisis point which effectively prevents health care systems from being able to reduce diabetes-related morbidity and mortality [9]. In 2017, there were 2.7 physicians and 12.4 nurses (including midwifery personnel) per 10,000 people in sub-Saharan Africa compared to 21.5 physicians and 44.9 nurses per 10,000 people in North America [9]. Although task-shifting from physicians to nurses is increasingly advocated as a potential solution to shortfalls in the number of physicians in sub-Saharan Africa, there is a striking paucity of data to support this approach [10]. Many studies [9,11-14] demonstrate the effectiveness of task-shifting, but what makes task-shifting effective in terms of diabetes self-management and in improving health outcomes is not understood, particularly in low-resource settings.

Given the myriad of barriers to the management of diabetes within sub-Saharan Africa and evidence from high-income countries that chronic disease interventions are most successful when implemented from a multidimensional perspective [15,16], it is anticipated that an effective intervention to improve type 2 diabetes disease management in sub-Saharan Africa will benefit from a novel approach that is founded upon solid theoretical constructs, built upon existing human resources and infrastructure, and culturally tailored to the priorities and needs of the local population. A recognized challenge in the successful implementation of a multidimensional approach within sub-Saharan Africa is that theoretical frameworks do not take into consideration health behaviors within the social and environmental constructs within which people live [11,15,17]. Available resources, such as mobile phones, can be used to help patients maintain glycemic control through health messaging and clinical reminders that are delivered by nurses who have been trained to manage additional day-to-day clinical responsibilities [11,17].

Given the aforementioned challenges, diabetes prevention and treatment strategies in sub-Saharan Africa must be culturally relevant; use a multidisciplinary approach that incorporates clinically directed management; shift day-to-day management of chronic care tasks from overextended physicians to trained nurses; capitalize on existing, low-cost resources such as mobile devices for ongoing, intermittent disease management; and be theoretically derived. An urgent priority in sub-Saharan Africa is, therefore, the improvement of health outcomes, self-management, and access to care for individuals with diabetes through programs that are theoretically guided and culturally relevant and that make use of task-shifting and available technology.

### Study Objectives

The objectives are to characterize the negative impacts of type 2 diabetes in sub-Saharan Africa and to prioritize the preferences of patients, caregivers, and health providers in the development of a theoretical, multilevel, culturally tailored nurse-led intervention that incorporates mobile health (mHealth) technology to increase treatment adherence, to improve outcomes, and to reduce the negative impact of diabetes in sub-Saharan Africa. Our mixed-methods protocol has the following goals:

1. Assess the characteristics (contextual factors, beliefs, practices, and self-management behaviors) of patients.

2. Assess the characteristics (beliefs, level of knowledge, access to, and familiarity with technology) of patients as well as technological barriers and facilitators, and their influence on outcomes at the various levels of the social-ecological model (individual, family/significant other/caregiver, health care organization, and community) using focused ethnography, a qualitative method.

3. To design a theory-based, multimodal nurse-led intervention that incorporates technology for diabetes management by converging quantitative and qualitative sets of data.

Our hypothesis is that a multilevel, culturally situated assessment of diabetes can be used to develop a nurse-led intervention and enhanced with mHealth technology for education and care management.

## Methods

### Study Setting and Population

The study will be conducted in Kumasi, Ghana which is among the largest metropolitan areas of Ghana and has a population of more than 2 million. The University Hospital, a district-level hospital located within the Kwame Nkrumah University of Science and Technology in Kumasi, will be the coordinating site for the study. The 100-bed hospital serves a population of over 200,000 people from more than 30 surrounding communities, treats approximately 86,000 outpatients each year, and has an active diabetes clinic that treats approximately 700 patients from diverse communities.

### Study Design

The study will employ a convergent parallel mixed-method design [18] and will be guided by the social-ecological model and community-based participatory research [19,20]. Quantitative data and qualitative data will be simultaneously collected, analyzed separately, and then integrated. Investigators will conduct focused ethnography, which has been described in previous literature and is useful for situation or problem-directed inquiry that allows for context-specific exploration among a subgroup [21,22], to explore nuances that are specific to patients with diabetes in Ghana in order to best inform the development of a tailored intervention that meets the clinical needs of patients with diabetes in sub-Saharan Africa.

Social-ecological model [19] constructs are used to explore the individual, interpersonal, and population health factors that are related to diabetes. This includes individual factors (genetics, pathophysiology, knowledge, attitudes, beliefs, and behaviors), interpersonal factors (family, friends, colleagues, peers, and support networks), organizational factors (health care institutions, faith-based organizations, workplaces, and schools), community (neighborhoods), and policies to achieve broad change (government and health care) which provide the overall framework for organizing our research and a lens through which the quantitative and qualitative data can be interpreted. Focused ethnography, in-depth interviews with patients and their caregivers, home and clinic observation, and rapid appraisal during interpersonal interactions with family, friends, and clinicians will be conducted.

### Study Procedures

Data will be collected from a convenience sample (N=100) of individuals with diabetes who are recruited from the hospital’s clinic. Surveys (described in Table 1) will use an interview format in their native language. Survey items assess demographics, diabetes self-management practices, beliefs, access to technology, attitudes toward mobile phone monitoring, and attitudes toward expanded management of care by nurses. Participant treatment beliefs are assessed through questions about their perceptions of treatment and about their understanding of diabetes. Health behavior will be assessed through survey items related to dietary habits, smoking, physical activity, adherence to treatment, and self-measured blood glucose testing. Participant understanding of long-term sequelae will be assessed (levels of risk associated with prolonged elevated blood glucose such as micro and macrovascular changes). Participant perceptions of the use of mHealth technology for the management of care and the use of nurses to allow increased access to day-to-day care will also be assessed.

**Table 1:** Survey descriptions.

Characteristics assessed	Survey description
1. Socio-economic	Descriptive demographic data—education, employment, and time since diabetes diagnosis
2. Access and preferences to technology	10-item survey assessing access to mobile health technology, technology preferences, and receptivity to intervention delivery using technology. This survey has undergone prior pilot testing in Ghana with a different study population [12] and was modified slightly for this study by changing stroke terminology to diabetes terminology.
3. Access to care	Assesses factors related to health care service access
4. Knowledge and practices related to diabetes care and disease progression	Assesses understanding of long-term effects of elevated blood glucose and diabetes self-care practices
5. Health habits	Inventory assessing current health habits
6. Social context	Qualitative, open-ended questions assessing individual, interpersonal, community, organizational, and policy-related factors including knowledge, attitudes, beliefs, practices, and preferences on diabetes treatment, diabetes care, health behaviors, mobile technology, and task-shifting.

A purposive study design using focus group discussions will assess characteristics, knowledge, attitudes, beliefs, and practices of Ghanaian individuals with diabetes. We will conduct interviews and observations in locations convenient to participants. Data will consist of field notes and video or audio recordings. Methods developed by Tremblay [23] will guide the interview format. Approximately 5 focus group sessions will be conducted, each with approximately 6 to 10 participants who are either patients, caregivers, community leaders, or faith-based leaders. Patients will be recruited from the survey group participants. After the survey, participants will be asked if they or their caregivers would be willing to participate in focus group discussions. Community and faith leaders will be recruited through referrals from local community partner organizations and, subsequently, their referrals. Focus group sessions will be conducted in a semistructured interview format to ensure systematic and consistent data on the following: beliefs about diabetes risk factor control, existing self-management practices and experiences, expectations and preferences, educational needs and preferences, impressions on nurse-led management of care and use of mHealth technology for remote monitoring, health reminders, or messaging. Separate focus groups will be held for community leaders in order to minimize inhibition in freely discussing ideas and thoughts. Patients and caregivers will be combined; these sessions are structured to maximize participant convenience with respect to transportation and scheduling. Focus group discussion prompts are tailored to general understanding of diabetes, current care practices and challenges, and preferences for future interventions. Since the individual roles and interpersonal relationships of patients and caregivers are not the focus, we hope to mitigate potential discomfort by openly sharing information across roles while maximizing convenience to participants. Focus group discussion prompt guides are detailed in Table 2.

**Table 2:** Focus group and interview discussion prompts.

Focus group composition	Discussion prompts
Patients and caregivers	What are some of the things you regularly do to help manage your (or your loved one’s) diabetes? What helps keep your blood glucose under control? What makes it difficult to control your blood glucose? What are some of the things you are worried about when it comes to your diabetes? Do you have any difficulty getting the care you need? If so, why do you think this is? What types of complications do you think you may have from your diabetes? What would you like to change about taking care of your diabetes? What types of things would you find more helpful to learn about? Tell me your thoughts on having a nurse help you regularly manage your diabetes? What are your thoughts about if the nurse communicated with you through the mobile phone? What else should we think about if we design a program to help people take better care of their diabetes?
Community and faith leaders	Do any of you have anyone in your community or home with diabetes? If so, what are some of the challenges people with diabetes face? As you all are community leaders, do people with diabetes come to you for any reason? If so, what types of things do they come to you for? What types of support, care, or services are they looking for? What do you think we can do to help people with diabetes in your community? Tell me your thoughts on potentially having a nurse help people with diabetes regularly manage their disease. What are your thoughts about using mobile phones to communicate with people with diabetes and help them manage their care? What challenges do you foresee with using mobile phones or having nurses help educate patients and manage their care? What recommendations do you have and what else should we consider that we haven’t asked about?
Key informant interview	How do you currently provide treatment for patients with diabetes? What are some of the challenges you and your patients face? What are some of the things that are working well? What do you perceive to be some of the main gaps in care and services for your patients with diabetes? What challenges do you face in communicating with your patients? What barriers exist, if any? Are the any cultural or spiritual considerations you need to take into account when providing care? What are your thoughts on the idea of task-shifting to a nurse-led model for day-to-day diabetes education and care management? What would you think is needed for education of the nursing personnel? What parameters should be considered in designing this type of care management system? What are your thoughts on the use of mobile health technology to manage diabetes? What are some considerations we should take into account in designing and planning for an intervention for diabetes care management in Ghana? What else is important for us to consider that we may not have asked about?

Individual interviews with key informants (health care providers and hospital administrators) will be conducted using a semistructured guide. Participants will represent Kwame Nkrumah University of Science and Technology departments (endocrinology, social work, nursing, pharmacy, and information technology). Interviews will include discussions on the following areas: current approaches to diabetes management, perceived gaps in care, cultural competence and communication, knowledge of treatment guidelines, impressions on nurse task-shifting and mHealth technology, and considerations or recommendations for intervention development (Table 2).

### Data Management and Analyses

Participants will be volunteers who have given written consent and will be free to withdraw at any time. All personal identifiers will be removed from any data and data will be analyzed and stored at the university on a secure network drive that is protected by password and used uniquely for this study. Access to the data will be limited to researchers involved in this study.

All data will be uploaded and stored on REDCap, a secure data storage system described in Harris et al [24]. Quantitative data will be analyzed using SPSS software (v24.0, IBM Corp). Focus group sessions and key informant interviews will be transcribed verbatim with any identifiers that might breach participant confidentiality redacted. For interviews conducted with non-English speaking individuals, transcriptions of audio recordings will be translated to English. De-identified transcripts of the audio recordings and field notes (including videos and photographs) will be used for qualitative data analysis. Transcripts will be verified using the recordings to confirm accuracy and authenticity. The transcripts will then be imported into the qualitative text analysis software (NVivo 12.0, QSR International) for data management and analysis. Qualitative data will be analyzed according to the social-ecological model to include inductive and deductive coding; theme development; identification of barriers, facilitators, knowledge, attitudes, beliefs, and preferences; perceptions of and recommendations on diabetes care, its management, mHealth technology, and task-shifting to increase care access and delivery. To maintain rigor within the qualitative data analysis, investigators will conform to standards of credibility, confirmability, transferability, and dependability [25,26]. Credibility will be maintained through prolonged exposure with data [13]. An audit trail of data collection and analysis will be used to confirm dependability of analytical processes [13], and researchers will account for personal bias [26]. Transferability and confirmability will be addressed through thick descriptions provided when reporting results, enabling clinicians and researchers to determine whether findings are consistent and transferable to their own sites or populations, and to have confidence that the interpretation of findings is derived directly from data [13]. Mixed methods allow comparison of data convergence and divergence; a matrix display will be developed for each construct of the social-ecological model (individual, interpersonal, organizational, community, and policy) to compare findings and assess convergence appropriately.

## Results

The study has been approved by the Committee on Human Research, Publications and Ethics at the Kwame Nkrumah University of Science and Technology (CHRPE/RC/241/17). The Medical University of South Carolina’s Institutional Review Board determined that, because the team at the Medical University of South Carolina would not be engaged directly with participants since they would be working only with de-identified data, Institutional Review Board approval was not required. Quantitative and qualitative data collection began in February 2018. Data analysis and the final report are expected to be completed by Spring 2020. As of May 2020, participant surveys have been completed (N=100), key informant interviews (n=7) have been completed, and focus groups (5 focus groups; patients, n=18; caregivers, n=6; community leaders, n=2; and faith leaders, n=3) as well as focused ethnographic field observations have been completed. All audio recordings have been transcribed and transcripts of sessions recorded in Twi have been translated to English. Data analysis is currently in progress. After separate quantitative and qualitative analysis, data will be combined for mixed analysis. When complete, results will be reported through professional presentations, peer-reviewed publications, and directly to Community Advisory Board members in Ghana.

## Discussion

The overall goals of this study are to develop a practical, collaborative, effective, and sustainable program for type 2 diabetes prevention and management in sub-Saharan Africa and to improve access to care through task-shifting and the use of technology. In addition, the proposed task-shifting strategy may mitigate the effects of the critical shortage in health care workers by allowing trained nurses to manage glycemic control in day-to-day care and by leveraging mobile phones that are already widely used in the region. To our knowledge, this would be the first study to establish a culturally sensitive, multilevel-systems effort to explore and document barriers and facilitators related to diabetes among Ghanaian patients; evaluate the knowledge, attitudes, and beliefs toward diabetes care management; identify access to technology and preferences related to mHealth technology as a mechanism for care delivery among Ghanaian patients with type 2 diabetes mellitus; develop a community-based intervention for patients with diabetes that will bridge the health system and community through a nurse-led model; and collect formative data from a diabetes intervention study in Ghana to use as a model for sub-Saharan Africa through focused ethnography. This study will inform the preliminary development of a feasible and effective mHealth intervention which may contribute to a definitive and more successful randomized control trial with the potential to reduce diabetes-related morbidity, mortality, and burden in sub-Saharan Africa.

## Abbreviations

mHealth: mobile health
NEEDS: Nurse-Led Education and Engagement Study for Diabetes Care
